# Bacterial morphotype grading for periodontal disease assessment

**DOI:** 10.1038/bdjopen.2016.11

**Published:** 2017-01-20

**Authors:** Kim Smeda-Pienaar, Eveline Kaambo, Charlene W J Africa

**Affiliations:** 1 Microbial Endogenous Infections Studies (MEnIS) Research Laboratories, Department of Medical Biosciences, Faculty of Natural Sciences, University of the Western Cape, Bellville, South Africa

## Abstract

**Background:**

Listgarten and Hellden (1978) used darkfield microscopy of wet mounts to differentiate between healthy and periodontally diseased sites in the mouth by expressing the different bacterial morphotypes observed as a percentage of the total number of bacteria counted. This method of periodontal disease assessment gained favour as a diagnostic tool but presented with the limitation of immediate examination to determine the number of motile rods present and an inability to distinguish between gingivitis and periodontitis. Grading of bacterial morphotypes into several distinct categories of health or disease (Ison and Hay, 2002), simplified the scoring system of Gram-stained smears for the diagnosis of bacterial vaginosis (Nugent *et al.* 1991). The application of a similar grading system using stained smears rather than wet mounts could be advantageous to the diagnosis of periodontal disease.

**Objectives/aims:**

This study tested the hypothesis that stained smears of dental plaque collected from the gingival crevice of individuals with varying probing pocket depths (PD) may provide a grading system for periodontal disease assessment.

**Materials and methods:**

Subgingival plaque samples were collected from 49 patients, stained with a silver stain and the proportions of each bacterial morphotype graded relative to their respective PD measurements.

**Results:**

This technique allowed for a grading system of I–IV, with grade I indicating health and grade IV indicating severe periodontal disease.

**Discussion:**

Stained smear examination eliminates the time restriction for motile rod enumeration and allows for storage of smears for future reference.

**Conclusion:**

Standardization of the microscopic areas to be evaluated or examined will facilitate the agreement of cut-off values for the diagnosis of periodontal disease.

## Introduction

Plaque-induced chronic inflammatory periodontal disease (CIPD) can be classified as gingivitis (gingival inflammation without loss of connective tissue attachment and alveolar bone) and periodontitis (with loss of the structures that support the tooth, namely, periodontal ligament, connective tissue attachment and alveolar bone). The clinical diagnosis of CIPD is therefore based on the periodontal probe measurements of periodontal pocket depth (PD) and clinical attachment loss (CAL). PD is measured from the gingival margin to the base of the probable crevice, while CAL is measured by as the distance from the cemento-enamel junction to the base of the probable crevice.^1^

Microbiological diagnosis of endogenous infections such as periodontal disease and bacterial vaginosis (BV) employ a system of microscopically counting bacterial morphotypes in wet mount preparations^2^ and Gram-stained smears,^3^ respectively, using cocci and increased motile rods and spirochaetes to differentiate between healthy and periodontally diseased sites in the mouth,^2^ and lactobacilli and mixed microbiota to differentiate between normal, healthy microbiota and BV-associated microbiota, respectively.^3^ Nugent *et al.* ^4^ improved on the BV scoring by introducing an intermediate category, characterised by mixed microbiota with a significant number of lactobacilli, while Ison and Hay^5^ simplified this scoring system by grading bacterial morphotypes into several distinct categories, thereby improving the diagnostic value of the Gram stain. Grade I consisted of lactobacilli only and was deemed normal. Grade II was deemed intermediate and consisted of mixed morphotypes with reduced lactobacilli. Grade III was considered to be indicative of BV, characterised by mixed morphotypes with few or no lactobacilli. The criteria of Listgarten and Hellden^2^ on the other hand, simply indicated health with cocci predominating and disease with motile rods and spirochaetes predominating with no categorisation of periodontal disease severity. Although favoured by other researchers as a reliable tool for assessing disease progression and/or response to treatment,^6,7^ the dark field technique has its limitations in that examination of the wet mount has to occur within an hour of preparation in order to evaluate the motile bacteria and often there was no association between what was observed microscopically and in culture,^6,8,9^ with few motile organisms and spirochaetes observed in culture. Nor was there a clear distinction between gingivitis and periodontitis. For this reason, it was considered that the application of a grading system for periodontal disease diagnosis, prompted by the grading system used by Ison and Hay,^5^ may favour further investigation to overcome the limitations of dark field microscopy of wet mounts.

### Objectives

This study tested the hypothesis that bacterial morphotype counts in stained smears of dental plaque collected from the gingival crevice of individuals with varying probing pocket depths (PD) may provide a grading system suitable for differentiating gingivitis and periodontitis.

## Materials and methods

### Sampling

Forty-nine patients attending a local periodontal clinic in the Western Cape, South Africa, were randomly selected for the study. The patients were aged between 25 and 45 years and were predominantly male.

Patients on antibiotics for 2 weeks preceding the study, denture wearers, pregnant women and diabetics or any other immunocompromised patients were excluded.

Supragingival plaque was removed with a scaler, and subgingival plague collected from 16 and 41 tooth sites with a sterile probe and dispersed in 0.5 ml 0.85% sterile saline solution (pH 7.4) in screw-capped vials.

A clinician recorded the periodontal condition of the patient by measuring in mm the probable (gingival) pocket depth (PD) from which the sample was collected.^10^

Ethics approval for the study was granted by the Research Committee of the University of the Western Cape and the study complied with the Declaration of Helsinki Principles.^11^ Informed consent for participation in the study was obtained and patients were assured of confidentiality.

### Microscopic examination

One drop (5 μl) of the vortexed plaque suspension was transferred to a glass microscopic slide, the slide was heat fixed by quickly passing it through a flame and stained with aluminium potassium sulphate/tannic acid/ferric chloride for 4 min, rinsed with running distilled water followed by staining with 5% silver nitrate by heating the slide until steam ensued and the slide left to stain for 4 min after removing the heat.^12^ Slides were air-dried after rinsing with running distilled water and examined with a light microscope at ×1,000 magnification. A total of 100 bacterial cells were counted from random fields and classified into morphotypes namely, spirochetes, motile rods (as indicated by the presence of stained flagella), other rods and cocci. For each slide, a duplicate count was performed from two different areas and the average score for each morphotype recorded. Each of the morphotypes was expressed as a percentage of the total number of cells counted. The microscopy was performed blind to the clinical scores recorded for each patient, thus eliminating the threat of bias. Charting of the clinical indices and the microscopy were done on separate pages, each coded with the patient number and matched after assessment.

Bacterial morphotype counts and PD were used to classify patients into Grades I to IV where Grade I indicated health, Grade II indicated an intermediate category, Grade III indicated established gingivitis and Grade IV indicated severe periodontal disease.

### Data analysis

The mean (s.d.) for each bacterial morphotype was obtained and recorded. Bar graphs were constructed using the mean and standard deviation values for each morphotype.

## Results

Using the silver staining method, bacterial morphotypes stained black against a white background. Table 1 depicts the grading system used to associate the relative proportion of bacterial morphotypes with their respective PD measurements.

Patients with PD<1 mm demonstrated high counts of coccoid cells and non-motile rods with few spirochetes and motile rods (Table 1). The mean value for cocci was 31%, but together with the other (non-motile) rods, they accounted for nearly 90% of the morphotypes counted. This morphotype distribution was designated Grade I.

Sites with PD 1–2 mm showed an increase in spirochaetes and motile rods with a decrease in cocci and non-motile rods (Table 1). This morphotype distribution and associated PD was graded as Grade II.

Grade III morphotype distribution showed a further increase in spirochetes and motile rods (Table 1) with decreased cocci and other rods which, associated with PD 3–4 mm, may indicate established gingivitis.^2,13^ A further increase in the spirochete count, with an average of 71% and a significant decrease in coccoid cells (*P*<0.05), motile and other rods was classified as Grade IV, which along with an associated PD ⩾5 mm, was consistent with severe periodontitis.^14,15^

**Figure 1:**
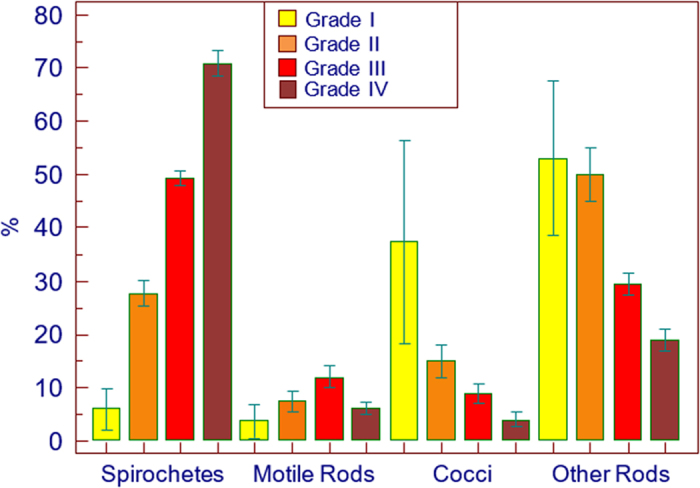
Bacterial morphotype distribution according to PD grading.

The changes in morphotype distribution for each grade are clearly captured in a bar graph (Figure 1), using the mean value of each morphotype in the four grades. Spirochaete counts increased significantly (*P*<0.05) from Grade I–IV while the reverse was observed for cocci (Figure 1).

Motile rods increased significantly (*P*<0.05) from Grade 1 to Grade III but dropped in Grade IV, while other rods followed the same pattern as the cocci, i.e., reducing significantly (*P*<0.05) in numbers from Grade I to Grade IV.

## Discussion

Detailed observation of the silver-stained bacterial morphotypes along with PD measurements facilitated the recognition of distinct grades of periodontal disease assessment from plaque smears.

Limitations of the darkfield microscopy technique include that results are only reliable if examined within an hour of collection, the reason being that bacteria lose their motility and can be erroneously classified as non-motile. The inability to differentiate bacterial morphotypes and Brownian movement from motility further adds to the complexity and can only be overcome through experience.^7^ Microscopic evaluation of dental plaque may provide valuable information regarding the disease status of an individual but is limited to the observation and reporting of bacterial morphology only, with no confirmation of the species present. Because there are significant differences among image areas covered by various microscopes, there could be a deviation in results obtained from different examiners.^16^

We elected to use the silver stain instead of the Gram stain, since spirochaetes are reported to be the predominant bacteria in periodontal disease and do not stain well with the Gram stain, thus they could be missed. Staining of bacterial smears has several advantages over dark field microscopy, namely, counting of immobilised bacteria is easier than that of motile bacteria and the flagella can be readily observed under light microscopy,^12^ thereby reducing the error of motility being confused with Brownian movement. Specimens do not have to be viable at the time of examination and the smears may be stored and examined at the convenience of the examiner. In addition, light microscopy is used, unlike dark field and phase contrast microscopy that employ special condensers.

Disadvantages of the staining method include indiscriminate precipitation in some areas, making it difficult to assess the morphotypes, retarded development of color (yellow instead of black) in some cases due to rapid cooling of the stain on the slide, and the excessive background produced when slides dry out due to prolonged staining.

Probing pocket depth (PD) and clinical attachment loss (CAL) are the most common clinical measures used in epidemiological studies for periodontitis with the periodontal probe being the most popular measurement tool used.^15^ In the present study, only PD measurements were used.

This grading system provided a scale of I–IV for the evaluation of subgingival plaque based on the percentage of 4 morphotypes namely spirochetes, coccoid cells, motile rods and other rods.

Sites with PD<1 revealed many cocci and non-motile rods consistent with reported normal oral microbiota in health, with few spirochaetes and motile rods. Grade I morphotypes therefore appear to be consistent with a healthy gingival crevice.

Grade II morphotypes (designated intermediate) appear to indicate an alteration in gingival status from health to the initial stages of gingivitis demonstrated by an increase in PD, spirochaetes and motile rods. We can speculate that this change in bacterial morphology may be due to the establishment of the capnophilic bacteria which sequentially colonise the oral biofilm after the facultative anaerobes have become established.

Spirochaetes are the predominant morphotypes microscopically observed in periodontal disease,^2,17^ but because of their fastidious growth requirements, they are often not detected in cultural studies. The results of the present study confirm their predominance in the Grades III and IV categories which, by PD measurements and observed morphotype counts, are suggestive of established gingivitis and severe periodontal disease respectively. Without radiographs, we were unable to confirm whether the Grade IV sites were indeed indicative of periodontitis. However, we have achieved our objective in establishing a microbial grading system for the interpretation of stained smears from varying pocket depths, in assessing periodontal disease severity.

Culture-independent molecular techniques have reported cocci such as *Gemella*, *Granulicatella*, *Streptococcus* and *Veillonella* with rods such as *Rothia dentocariosa* and *Actinomyces* as the predominant bacteria in normal healthy microflora of the oral cavity.^18,19^ This could be indicated by the Grade I morphotypes viz increased cocci and rods observed microscopically in this study in patients with PD<1 mm. Grade II morphotypes indicated increased spirochaetes and motile rods with an increase in PD from 1 to 2 mm demonstrating a transient phase, with a change in the ecology from health towards disease. Grade III morphotypes with an increase in spirochaetes and motile rods and a concomitant decrease in cocci and non-motile rods in sites with PD 3–4 mm could indicate an established gingivitis. Motile rods such as the capnophilic Gram-negative bacteria *Capnocytophaga, Selenomonas* and *Wolinella* are known to predominate in gingivitis lesions while other rods such as *Actinomyces* and *Eubacteria*, may or may not display motility and could be captured in either category. *Selenomonas* species and *Dialister* species have been associated with a worsening periodontal status.^20^ These species appear in the oral biofilm just before the established climax community of Grade IV morphotypes namely, increased spirochaetes and non-motile rods (or cocco-bacilli) resembling *Porphyromonas*, *Tannerella*, *Prevotella*, *Bacteroides*, *Aggregatibacter actinomycetemcomitans (Aa)*, *Dialister* and *Fusobacterium.* ^21,22^

## Conclusion

Stained smear examination has an advantage over wet mount examination in that it eliminates the time restriction for motile rod enumeration, while also allowing for the storage of stained smears for future reference.^12^ Standardisation of the microscopic areas to be evaluated or examined will facilitate the agreement of cutoff values for the diagnosis of periodontal disease. While the authors take cognizance of the fact that the small sample size of this study may be a limitation, the results presented in this study look promising and warrant further application.

## Figures and Tables

**Table 1:** Grading system associating bacterial morphotypes with PD

*Grade*	*% Spirochetes mean (s.d.)*	*% Motile rods mean (s.d.)*	*% Cocci mean (s.d.)*	*% Other rods mean (s.d.)*	*PD (mm)*
I (*n*=4)	9 (8.04)	4 (4.5)	31 (30.4)	57 (18.9)	<1
II (*n*=8)	29 (6.5)	8 (5.7)	16 (9.5)	48 (14.2)	1–2
III (*n*=17)	49 (6.1)	12 (8.7)	9 (7.5)	29 (8.5)	3–4
IV (*n*=20)	71 (10.4)	6 (5.5)	4 (5.9)	19 (9.1)	⩾5

Abbreviation: PD, pocket depths.
